# A Facile Determination of Herbicide Residues and Its Application in On-Site Analysis

**DOI:** 10.3390/foods13081280

**Published:** 2024-04-22

**Authors:** Yifei Sun, Yan Tang, Zetao Chen, Miaoxiu Ge, Wei Xiong, Luhong Wen

**Affiliations:** 1 The Research Institute of Advanced Technology, Ningbo University, Ningbo 315211, China; 2111082241@nbu.edu.cn (Y.S.); tangyan@nbu.edu.cn (Y.T.); 2211090085@nbu.edu.cn (Z.C.); 2211390101@nbu.edu.cn (M.G.); wenluhong@nbu.edu.cn (L.W.); 2 Faculty of Electrical Engineering and Computer Science, Ningbo University, Ningbo 315211, China; 3 China Innovation Instrument Co., Ningbo 315100, China

**Keywords:** food safety, herbicides, crops, on-site analysis, method validation, ionization of analyte

## Abstract

Abuse of herbicides in food safety is a vital concern that has an influence on the sustainable development of the world. This work presents, a modified ionization method with separation of the sample and carrier gas inlets, which was utilized for efficient ionization and analyte transfer of herbicides in crops. The working parameters of voltage, injective distance, desorption temperature, and the carrier gas flow rate were optimized to achieve the high efficiency of the transfer and ionization of the analyte. When it was applied in the analysis of herbicides in laboratory, the method exhibited excellent performance in achieving the quantitative detection of herbicides in solutions and residues spiked in an actual matrix with a limit of quantification of 1–20 μg/kg and relative standard deviations of less than 15%. Although a simple QuEchERS process was used, the programmable heating platform ensured efficient gasification and transfer of the target analyte, with the advantages of high speed and selectivity, avoiding the noted matrix effect. The method exhibited a relatively acceptable performance by using air as the discharged gas (open air). It could be used to monitor herbicide residues in the growth stage via on-site non-destructive analysis, which obtained low LODs by dissociating the herbicides from the crops without any pretreatment. It showed great potential for the supervision of the food safety market by achieving non-destructive detection of crops anytime and anywhere. This finding may provide new insights into the determination of pesticide emergence and rice quality assessment.

## 1. Introduction

Food safety is a vital concern in the 21st century, as it has a serious influence on the sustainable development of the world [1]. In order to increase crop yields by avoiding nutritional competition between crops and weeds, triazine pesticides (TPs), carbamate pesticides (CMs), and amide pesticides are widely used in agricultural production as typical representatives of efficient herbicides [2]. These kinds of herbicides are used in isolation or in combination during the period of rice crops’ growth, resulting in the same effect as weeding [2]. However, herbicide residues are a universal phenomenon in agricultural production, where the process of spraying herbicides may lead to the absorption of herbicides into crops, resulting in indirectly effects on human health [1,3,4]. Due to the abuse of these herbicides, their residues have been found in rice crops and the surface water near rice paddies, which had concentration ranges of 5–270 μg/kg and 0.2–340 ng/L, respectively, creating a threat to health. Based on the regulatory standards of the EU or China, pesticides need to be correctly dispensed and applied to reduce the impact of pesticide residues on the human body and the environment. Nowadays, liquid chromatography (LC) [5,6] and gas chromatography (GC) [7,8] coupled with triple quadrupole mass spectrometry, which have the function of a multiple reaction monitoring (MRM) mode, are the most widely used techniques for quantitatively analyzing herbicide residues in foods [9]. These professional instruments need a strict pretreatment process and frequent maintenance of the ion source for protecting delicate parts from low detection efficiency that cannot meet the requirements of large-scale detection. Thus, it is of great importance to develop an efficient, rapid, and accurate screening method to satisfy the demands of large-scale quantitative screening [10]. Moreover, real-time and on-site detection of herbicides with convenient or no sample pretreatment, and which avoids the chromatographic separation step, can save costs in daily operation but remains a challenge [11]. This convenient and practical approach, which fulfils many green analytical chemistry principles, is made feasible by using ambient MS techniques.

Over the past decade, ambient mass spectrometry (AMS) has become an important scientific research tool in various important research fields, such as pharmaceuticals, food, environment, public safety, and clinical diagnosis [12]. Ambient ionization (AI) technology, as the key to AMS analysis, allows the direct sampling/ionization of analytes from raw practical samples with simple or no sample pretreatment under open atmospheric pressure environments, and has greatly improved the analytical efficiency [13]. With the advent of desorption electrospray ionization (DESI) [14,15,16] and direct analysis in real time (DART) [17], many kinds of atmospheric ion sources have been proposed, such as dielectric barrier discharge ion source (DBDI) [18], desorption atmospheric pressure chemical ionization (DAPCI) [19], and extractive electrospray ionization (EESI) [20], etc. Dielectric barrier discharge (DBD) is a discharge technique that generates stable low-temperature plasma at atmospheric pressure [21,22], which was first introduced into mass analysis by Zhang’s group [18]. In accordance with the discharge principle, two discharge electrodes and an insulating medium (e.g., quartz glass) are needed [23]. After long-term application in many fields, the advantages of this type of ion source can be summarized as follows: (1) a wide application range satisfying the ionization of molecules with relatively low polarity; (2) strong ability to ionize analytes which can achieve high ionization efficiency on complex samples with a reduced matrix ion suppression effect [24].

Unlike the formerly reported structure of DBD [25], a sample tube that acted as sampling and an insulating medium coupled with electrodes was constructed for AMS analysis [26,27,28]. The analytes could be directly transported through the sample tube into the mass spectrometer via the pressure difference between the atmospheric environment and the front vacuum chamber, where the analytes were simultaneously ionized [29]. This structure for simultaneous ionization and ion transportation could help to achieve higher sensitivity, because ionization occurred inside the source rather than in an open environment [30]. Although this ion source exhibited excellent performance in the detection of gas samples, ionization of the analytes might be influenced by the collision efficiency of the plasma and analytes, which had the same flow direction to the MS inlet. Moreover, long-term performance might gradually decrease because of the accumulated adhesion of analytes to the electrode, limiting its application in complex samples [31]. In consideration of its application in the detection of many kinds of herbicides, such as the triazine-type (e.g., prometryn), carbamate-type (e.g., molinate), and acetylaniline-type (e.g., acetochlor, pretilachlor), each of herbicides has different physicochemical properties to give different dissociative and ionized parameters, resulting in limitations in the simultaneous detection of a broad class of herbicides. Therefore, constructing an ion source coupled with a thermal desorption platform and a separate sample inlet to enhance the efficiency of ionization and long-term stability is urgently needed but remains quite challenging.

**Figure 1:**
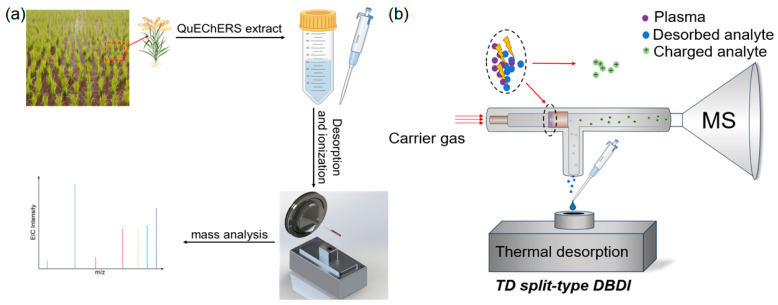
(**a**) Photographs of the analytical procedure using this method in the laboratory (different colors in the mass spectrum represent different ions). (**b**) Schematic illustrations (not proportional) of the desorption and the ionization process. Analytes in their liquid or solid states are desorbed via programmable thermal heating, where the desorbed analytes entered the sample tube to react with the plasma to form analyte ions and enter the MS inlet for detection.

In this work, we present a novel dissociated and ionized device that is a combination of a programmable heat platform and DBDI with separation of the sample and gas inlets (named the TD split-type DBDI), achieving sensitive detection of a broad class of herbicides (Figure 1). We demonstrated that the design of the inlet separation gives a great boost in the ionization efficiency and long-term stability, ascribed to the high-efficiency collision and little adhesion to the electrode. For different kinds of herbicides, analytes can be simultaneously detected by synchronous heating dissociation and ionization within the ion-transporting tube, giving high detection sensitivity [32]. When it was applied in the laboratory analysis, the sample was pretreated by a simple QuEchERS process to extract the liquid, which was directly dissociated and ionized, achieving a limit of quantification (LOQ) of 1–20 μg/kg. Furthermore, for on-site detection, a piece of spike or leaf from rice (1 cm) was placed onto the dissociative platform to generate gaseous molecules that were efficiently ionized, thereby achieving excellent sensitivity. This technique could be used to detect low-volatile and non-volatile chemicals, and has a great potential in multiple application scenarios such as in laboratory and on-site analyses.

## 2. Materials and Methods

### 2.1. Chemicals and Materials

Methanol (HPLC grade) was purchased from TEDIA Co. (Fairfield, OH, USA). Helium (99.99%) and nitrogen (99.99%) from Fangxin Gas Co. (Ningbo, China) were used as the carrier gases. Standard substances such as alachlor, acetochlor, pretilachlor, butachlor, promethazin, and dichlorpyr in methanol (10 ppm) were purchased from Aladdin Regent Co. (Shanghai, China). The relatively diluted solutions of standard substances were obtained by adding the relative volume of methanol. Extraction packages (QuEChERS Extract Pouch EN Method) were purchased from Biocomma Co. (Shenzhen, China).

### 2.2. Instruments

The pipette gun (10 μL) used for injection of the sample was purchased from Eppendorf Co. (Hamburg, Germany). The programable thermal desorption (TD) platform and programable software were manufactured by Ningbo Hua Yi Ning Chuang Intelligent Technology Co. (Ningbo, China). The adjustable voltage power supply with a range from 1 to 5 kV was purchased from CORONA LAB. (Nanjing, China). The ion source was laboratory-made, which consisted of a T-type quartz glass and two electrodes (a copper rod and gold foil were used as the inner and outer electrodes, respectively). The simulation was completed by COMSOL Multiphysics^®^ from COMSOL Inc. (Stockholm, Sweden). The ion source device was simplified as a T-shaped tube, and the mass spectrometer injection port was connected with a circular quartz tube with a length of 42 mm and a width of 2 mm. The diameter and height of the injection pipe were set to 1 mm and 10.5 mm, respectively. The pressure of the mass spectrometer’s injection port was replaced by the prestage vacuum chamber in the MS, and different simulation results were obtained by changing the flow rate of the carrier gas.

Molecular ions were analyzed by using a commercial linear ion trap mass spectrometer (Scientific Finnigan LTQ, Thermo Fisher Scientific, San Jose, CA, USA). Then the obtained data were processed via the instrument’s software (Xcalibur version 1.4 SR1) to obtain the ion’s information and standard curves. The running conditions of the mass spectrometer were set as follows: tube lens voltage, 115 V; capillary temperature, 275 °C; multipole RF amplitude (Vp-p), 400 V; multiplier voltages 1 and 2, −1200 V. The ion injection time was set to 100 ms, and the number of microscans was set to one. The performance of the mass spectrometer was optimized according to the operation manual from Thermo Fisher Scientific.

The volume deposited for desorption was 10 µL in all the experiments, unless otherwise specified.

### 2.3. Sample Preparation and Treatment

For pretreatment of the sample, the EN-15662 QuEChERS procedure was used [33], and methanol (10 mL) was used as the extraction solution. The rice samples (such as grains and straw) were homogenized using an electric pulverizer for subsequent experiments. The crushed rice samples (10 g ± 0.001 g) were transferred to 50 mL plastic centrifuge tubes, and some volumes of the relative concentrations of standard alachlor, acetochlor, pretilachlor, butachlor, prometryn, and molinate (in methanol) were added to obtain matrix-contained standard samples. For example, adding 100 μL of a 10 ppm standard solution into crushed rice samples (10 g ± 0.001 g) obtained matrix-contained spiked samples (100 μg/kg). Five or six spiked levels for each herbicide were used in this work. For example, the spiked concentrations of 1, 5, 10, 20, 50, and 100 μg/kg were selected for validation of the analytical method with prometryn. The detailed preparation method of the matrix-contained standard samples with different concentrations is shown in Table S1. The samples were vortexed for 1 min to completely disperse the standards in the matrix. Then an extraction package was directly added to each centrifuge tube, which contained 4 g of anhydrous MgSO_4_, 1 g of NaCl, 1 g trisodium citrate dihydrate, and 0.5 g of disodium hydrogen citrate sesquihydrate (MgSO_4_ was used to eliminate excess water; other buffer salts can adjust the pH value of the sample solution to ensure better recovery rates for alkaline-sensitive pesticides). Then the mixture was shaken vigorously for 1 min and centrifuged at 5000 rpm for 3 min. Finally, the supernatant was separated to be used directly as a sample solution for the subsequent MS analysis.

## 3. Results and Discussion

### 3.1. Optimization of the Parameters

According to the routine analytical process, five separate analytical steps, namely sampling, desorption, ionization, ion analysis, and platform cleaning, are involved in the process of TD split-type DBDI-MS analysis. In order to obtain the optimal parameters for the TD split-type DBDI-MS analysis system, we chose pretilachlor as a verified substance, which had a parent ion *m*/*z* of 312.2 and a fragment ion *m*/*z* of 252.2 (Figure S1). In this experiment, the added volume of the standard solution was 10 μL. The whole analytical process for a single sample was operated under atmospheric pressure, where the total time including the dissociation and analysis was less than 10 s. In addition, to avoid cross contamination, the pipetting head was replaced after a single use. The residues on the thermal desorption platform were washed using methanol and acetonitrile solutions in sequence.

**Figure 2:**
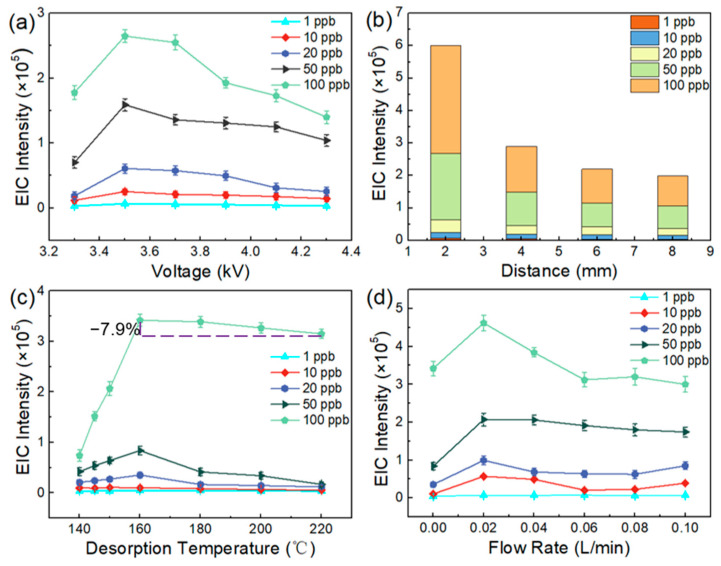
The changes in the EICs’ intensity for pretilachlor’s parent ion (*m*/*z* 312.2) at various concentrations depending on (**a**) the ion source’s voltage setting, (**b**) the distance between the thermal desorption platform and the sample injection port, (**c**) the desorption temperature, and (**d**) the flow rate of the carrier gas (*n* = 3; the error bar represents the standard deviation of three tests).

As the literature has previously reported [34], a high voltage for the generation of plasma is a key parameter for the ionization efficiency of a DBD-based ion source. When the onset voltage (i.e., 3.3 kV) for generating the plasma was reached, the analyte’s ion signal was obtained. The optimal setting for the voltage was acquired by evaluation of the intensity of the target ions. When the voltage setting ranged from 3.3 to 4.3 kV, fragment ion signals of different concentrations of the analyte showed a similar variation trend, in which the highest ion signal was obtained under the voltage conditions of 3.5 kV (Figure 2a). This phenomenon might be ascribed to the high-energy plasma generated by a higher voltage, which might reduce the formation of molecular ions [35]. In terms of the ambient ion source, the efficiency of ion transportation plays an important role in obtaining excellent sensitivity [36], hence the modification of the inlet’s design was needed. As a prerequisite of a normal sample, the influence of the injection distance between the top of the thermal desorption platform and the sample injection port was investigated. As shown in Figure 2b, when we added different concentrations of pretilachlor onto the TD platform, all the added samples obtained the highest ion signals while the injection distance was set to 2 mm, which demonstrated that this distance was suitable for efficient transportation of gases and detection as well. After completing the optimization of the basic hardware for the TD split-type DBDI system, the desorption temperature and flow rate of carrier gas (helium) were taken into consideration, which might affect the degree of formation of the gaseous analyte’s molecules, the efficiency of transportation to the DBDI source, and the ionization efficiency. As shown in Figure 2c, when the desorption temperature was set to 160 °C, the ion signal’s strength was the highest, ascribed to the formation of a spray-like mechanism. However, as the temperature continued to rise, it was found that the ion signal’s strength decreased, which resulted from the Leidenfrost phenomenon that preventing the liquid from absorbing heat [37]. At the same time, the liquid bounced irregularly on the hot surface, leading to a reduction in the amount of the sample’s molecules that entered the injection port. Moreover, the flow rate and type of carrier gas are important parameters for the ionized capability of DBDI [38]. However, for relatively high concentrations (e.g., 100 ppb), the effect of the Leidenfrost phenomenon may be in competition with volatilization, which produced a slight decrease in the signal’s strength. The effect of the flow rate of the carrier gas on the ion signal was studied. The results in Figure 2d show that the existence of helium gas at 0.02 L/min gave the highest ion signal, indicating that collision of the ions and ionization were most effective under these conditions. Moreover, when the carrier gas was air, namely when the flow rate was 0 L/min, relatively strong ion signals were still achieved, which indicated that the TD split-type DBDI could work normally under an air atmosphere. As the pressure of the prestage vacuum chamber in the MS was 0.9 Torr, the amount of gas in the gas inlet would have negative effects on the sampling and working status of the MS, wherein dissociative gaseous molecules could not be inhaled into the ionization tube, resulting in inefficient collision between plasma and gas analyte (Figure 2d). This demonstrated that the airflow balance between the sample and the carrier gas inlet had an impact on the possibility of a collision between the particles and the sample, which may have determined the ionization and transportation efficiency. In summary, the parameters with a voltage of 3.5 kV, a flow rate of the carrier gas of 0.02 L/min, and a desorption temperature of 160 °C were selected for the subsequent validation of the method on various herbicides unless specified.

**Figure 3:**
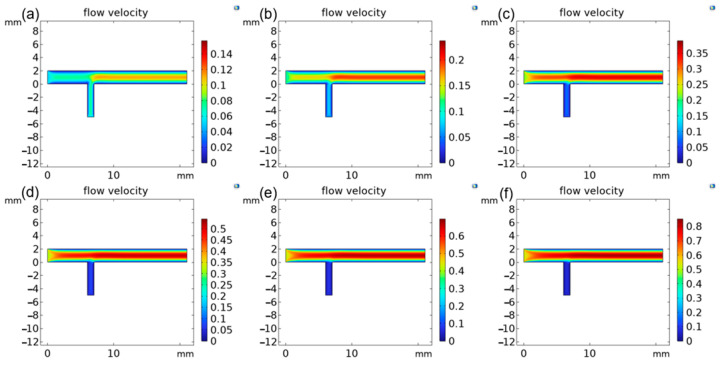
Simulations of the densities of the carrier gas and sample gas when the velocity of the carrier gas was set to: (**a**) 0 L/min (atmospheric), (**b**) 0.02 L/min, (**c**) 0.04 L/min, (**d**) 0.06 L/min, (**e**) 0.08 L/min, and (**f**) 0.10 L/min.

According to the confirmatory experiments above, the flow rate of the carrier gas that acted as a discharge gas for the DBDI source was conducive to ion transport and ionization of the analyte, which contributed to a decrease in the analyte’s adhesion to the inner surface. Helium, as an inert gas, was used for generating the plasma beam that participated in the ionization process to increase the probability of molecular ionization, where the carrier gas activated the ionization process and efficiency by enhancing the collision of particles [39,40]. As we all know, the pressure in the inlet of a MS is an unaltered value, which means that amount of gas intake from the inlet of the MS is equal per unit of time. When the carrier gas inlet has a larger gas intake, the partial pressure in sample inlet is small so that major gaseous analytes cannot be inhaled into the tube. Furthermore, this non-uniform distribution of pressure brings out an inadequate dynamic interaction between the plasma and the gaseous analyte, resulting in lower ionization efficiency and utilization of the sample. On the other hand, the high flow rate of the carrier gas increases the ionization of gaseous impurities in the environment, resulting in enhancement of the background base signals. Importantly, the carrier gas influences the spectral peak and ion separation efficiency [41]. In order to thoroughly understand the flow rate of the carrier gas that affects the particle collisions between the plasma and analytes, we used the function of gas simulation in COMSOL Multiphysics to see the flows of the carrier gas and the gaseous analyte inside the ionization tube when using a certain flow rate of the carrier gas. Six flow velocities of the carrier gas (0 L/min, 0.02 L/min, 0.04 L/min, 0.06 L/min, 0.08 L/min, and 0.10 L/min) were chosen to investigate the densities of plasma generated inside the tube and the gaseous analyte inhaled into tube. As shown in Figure 3, the velocity of the sample at the inlet decreased with an increase in the velocity of the carrier gas, which caused a reduction in the probability of a collision in the section of the two inlets to affect the signal intensities of the target ions (Figure 2d). Thus, a flow rate of the carrier gas of 0.02 L/min was chosen for giving a balance between ionization efficiency and the efficiency of transferring the sample.

### 3.2. Validation of the Method in the Detection of Herbicides

Having obtained an ionization system with excellent performance, we investigated the optimized ionization system applied for the quantitative detection of different kinds of herbicides. Different kinds of herbicides, namely alachlor, acetochlor, pretilachlor, butachlor, prometryn, and molinate, were chosen as targets of the analysis. Their detailed information is summarized in Table S2. To achieve better quantitative experiments, full scanning and CID experiments were utilized to identify the herbicides in methanol at a concentration of 10 ppb. The experiments above were conducted by the modified ionization system (TD split-type DBDI) in this work. The molecular ions [M+H]^+^, their MS/MS spectra, and fragment ions of the six kinds of herbicides are shown in Figures S1–S6. According to the intensities of the fragment ions and previous literature [42], all of the quantitative ions were selected and are listed in Table S2.

Under the optimized analytical parameters, the standard correction curves for each herbicide were constructed by plotting the signals’ intensity (the integration of the extracted ion chronogram (EIC) of each quantitative ion) versus the concentrations of herbicides. The linear range, regression equations, and linear correlation coefficients (R^2^) are shown in Figure S7 and Table S3. The LODs of all analytes were determined by a signal-to-noise (S/N) ratio of 3. The LOQs of all analytes were determined by a signal-to-noise (S/N) ratio of 10. Precision refers to the degree of closeness between the results obtained by multiple injections under specified testing conditions, which was expressed as the relative standard deviation (RSD). Accuracy refers to the closeness of the result measured by the established method to the true value, which was expressed as the recovery. As the results depicted, the TD split-type DBDI system with optimized parameters could provide low LODs of 0.03–1.5 ppb with RSDs of less than 15% and R^2^ values of 0.9856–0.9980 for all six analytes. The modified ion source coupled with the mass spectrometry method applied in the detection of herbicides exhibited many advantages, including high sensitivity, high speed, and convenience for the detection of herbicides.

### 3.3. Analytical Performance in Spiked Samples

**Figure 4:**
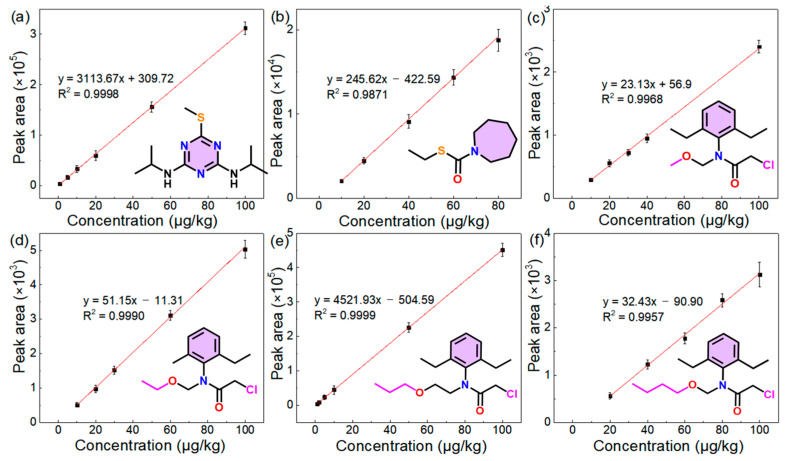
Standard calibration curves for six herbicides spiked in rice samples at various concentrations of 1–100 μg/kg: (**a**) prometryn, (**b**) molinate, (**c**) alachlor, (**d**) acetochlor, (**e**) pretilachlor, and (**f**) butachlor (*n* = 3; the error bar represents the standard deviation of three tests).

The analytical performance of this detection system was investigated by detecting six kinds of herbicides spiked in rice samples (e.g., leaves and spikes, etc.). The addition of different amounts of herbicides in the spiked samples was performed by the method described in Section 2.3; for example, injecting a standard solution of 10 ppm (100 μL) into a pretreated rice sample (10 g) obtained a spiked sample with a mass concentration of 100 μg/kg. Concentrations of 1–100 μg/kg for rice samples spiked with six kinds of herbicides were chosen to construct standard correction curves by plotting the signal’s intensity (the integration of the EICs) versus the concentrations of the spiked samples; simultaneously, the parameters of the linear range, regression equations, R^2^, and RSDs were obtained. As shown in Figure 4 and Table 1, LODs of 0.3–6 μg/kg with RSDs of less than 15% and R^2^ values of 0.9871–0.9999 for all six analytes in the spiked samples were obtained. All recovery values met the acceptability requirements (recovery: 63–97%). Although the quantitative detection results of spiked the samples exhibited a small matrix-effect that might reduce the precision of quantitation compared with that of the standard substance solution (Figure S7), the deviations in the quantitation by the AMS method in this work could be tolerated because of the adequate sensitivity and detection speed.

Based on the requirements of the national food safety standard of China (GB2763-2021), the allowable daily intake (ADI) of alachlor, acetochlor, pretilachlor, butachlor, prometryn, and molinate in food is 10, 10, 18, 100, 40, and 1 μg/kg, respectively, and the maximum residue limits (MRLs) of them in food are 50, 50, 10, 100, 20, and 100 μg/kg, respectively. On basis of the detection data, the LODs of the detection of herbicide residues in actual rice samples were confirmed to satisfy the associated national standard, suggesting that the modified TD split-type DBDI system could provide support for the supervision of food safety. Overall, this proposed method offers a new concept that has potential for rapid analysis and a cost-effective device compared with other methods.

### 3.4. Simultaneous Analysis of Herbicides Spiked in Rice Samples

**Figure 5:**
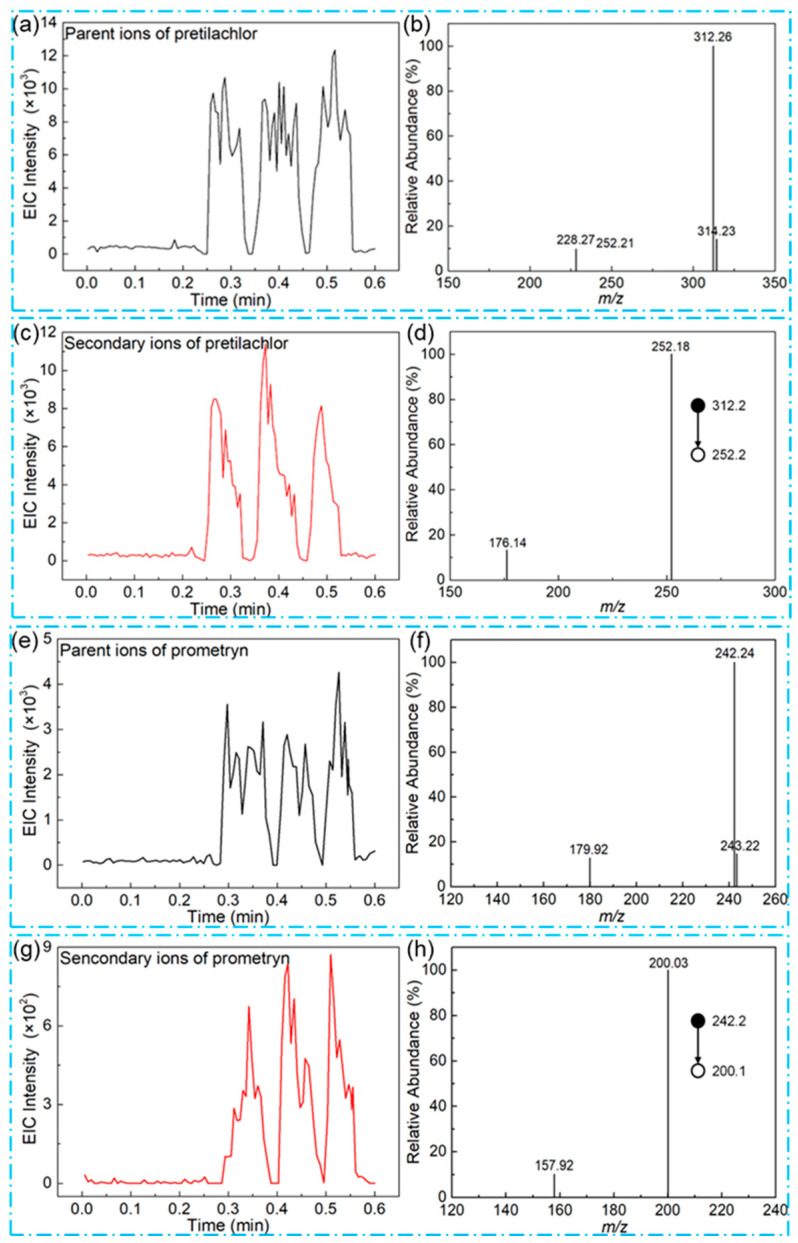
The EIC peaks for the parent and fragment ions of pretilachlor (**a**,**c**) and prometryn (**e**,**g**) in 5 μg/kg spiked rice samples with three consecutive injections, and the corresponding mass spectra of pretilachlor ((**b**) *m*/*z* 312.2 for the parent ion, (**d**) *m*/*z* 252.2 for the fragment ion) and prometryn ((**f**) *m*/*z* 242.2 for the parent ion, (**h**) *m*/*z* 200.1 for the fragment ion).

As the pretreatment process was simple, it might cause many matrices to disperse into the extract. A programmable heat platform was used to convert the analyte into the gaseous stage for direct inhalation, while preventing many substances with a large molecular weight (e.g., pigments and cellulose, etc.) from being inhaled, reducing competitive ionization. Direct online analysis of the extracts of the samples is important for identifying the existence of hazards in crops and is beneficial for the supervisor to give early warning [43]. Therefore, to further demonstrate the capability of the TD split-type DBDI applied in the direct detection of herbicides in complex samples, analyses of the fragmentation ion EICs from the six herbicides with different spiked concentrations were performed. As shown in Figure 5a,b, the EIC peaks with three consecutive injections for the parent ion of pretilachlor were clear and sharp, and the corresponding mass spectrum of one injection appeared for an ion peak of *m*/*z* 312.26 as the predominant peak accompanied by several impurity peaks despite the lack of chromatography. Furthermore, the results including the EICs’ peaks with three consecutive injections and the corresponding mass spectra for the fragmentation ion of *m*/*z* 252.18 of pretilachlor were obtained (Figure 5c,d, respectively), which exhibited the same MS/MS spectrum as standard pretilachlor (Figure S1). Next, an analysis of the EIC and the corresponding MS spectra from prometryn was performed following the same protocol as pretilachlor, which gave concise EIC peaks and almost perfect MS spectra compared with the standard substance (Figure 5e–h). On the basis of the findings above, the TD split-type DBDI method for the spiked samples with alachlor, acetochlor, butachlor, and molinate was used to achieve the detailed data of the EIC and MS spectra. The results showed that all EIC peaks gave quick responses, and the MS spectra were standardized with only small impurity peaks, which were consistent in those of pretilachlor and prometryn (Figure S8). Interestingly, the EICs of all spiked rice samples had clear and sharp shapes indicative of the quick gasification and transfer of the analytes. Moreover, the MS spectra of each targeted analyte showed that the molecular ion peaks had a high abundance, even when negligible impurity peaks existed, which gave a further demonstration that the programmable ionization method could selectively let the analytes gasify, while avoiding a large amount of the matrix in the ionized region. Considering the use of a small injection volume (10 μL), the solvent effect could be neglected.

Therefore, the TD split-type DBDI approach provides a new method for the sensitive detection of herbicides in actual crops, which exhibits great advantages in the gasification, ionization, and transfer of analyte, as well as partly reducing matrix effects.

**Figure 6:**
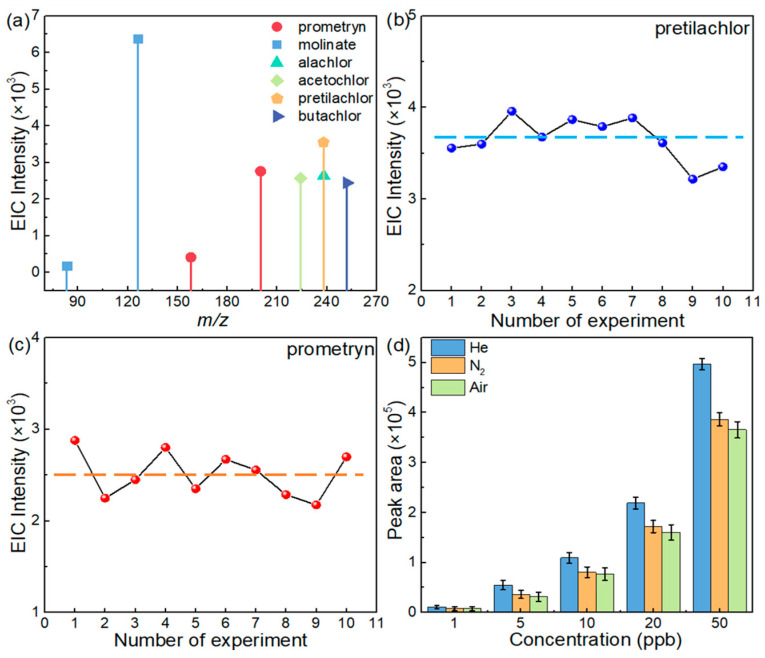
(**a**) The EICs’ intensities of rice spiked with a mix of six herbicides at concentrations of 5–50 μg/kg in spiked blank extracts of rice. The change in the signal’s intensity of one injection was determined each time after 10 experiments: (**b**) pretilachlor and (**c**) prometryn at 1 μg/kg in spiked rice samples (The blue and orange spheres represent the signal strength of pretilachlor and prometryn in each experiment). (**d**) The peak area of the EIC peaks for pretilachlor ions (*m*/*z* 312.2) at various concentrations, when using three different kinds of carrier gas as the discharge gas (*n* = 3; the error bar represents the standard deviation of three tests).

Actually, more than one kind of herbicide exists in crops. It is necessary to develop a rapid detection method for the simultaneous detection of different herbicides. The performance in detecting six different herbicides of the proposed method in this work was investigated. Six different herbicides, including molinate (2 ppm, 100 μL), prometryn and pretilachlor (0.5 ppm, 100 μL), alachlor, acetochlor and butachlor (5 ppm, 100 μL), were added to homogeneous rice (10 g) to prepare spiked samples, and the samples with spiked herbicides (molinate, 20 μg/kg; prometryn and pretilachlor, 5 μg/kg; alachlor, acetochlor, and butachlor, 50 μg/kg) were pretreated by the general method mentioned in Section 2.3. After obtaining the treated supernatant, the analytical process followed the method above. As shown in Figure 6a, the MS/MS spectra of molecular ions with a strong EIC intensity and sharp peaks (Figure S9) demonstrated that all herbicides could be simultaneously detected and efficiently analyzed, which could meet the general needs of the national standard (10–100 μg/kg, Table 1). The average EICs’ intensities of the fragmentation ions for the six herbicides with three consecutive injections are summarized in Figure 6a, giving an intuitive understanding of the detective performance. It is worth noting that alachlor and acetochlor are isomers, as are pretilachlor and butachlor. It is difficult to distinguish them by primary scanning via low-resolution mass spectrometry, while using MS/MS technology could achieve significant distinctions from each other, because of the different secondary ions (Figures S1, S3, S4 and S6). Furthermore, stability and repeatability were important evaluation parameters of the TD split-type DBDI system. We conducted multiple repeat experiments by using pretilachlor- and prometryn-spiked rice samples (1 μg/kg). Specifically, we performed the experiments 10 times under the same experimental conditions and operational procedures. As shown in Figure 6b,c, the EIC’s intensities for the fragmentation ions from pretilachlor and prometryn were collected, and the variative curves were constructed by plotting the EIC’s intensity for one injection versus the number of experiments. By a further analysis of the curves, the RSDs of the curves for pretilachlor and prometryn were 6.50% and 9.78%, respectively. After the experiment was run 10 times, the ion intensity of the analytes maintained almost the same intensity as that of the first time, which verified that the TD split-type DBDI ionized system had good stability and anti-pollution capacity.

Moreover, the site conditions may play an important role in on-site rapid detection, a cylinder of helium gas cannot be easily carried. Hence, it is of great significance to stimulate the development of an ionized system without a carrier gas. We chose pretilachlor as analytical model to conduct the experiment that tested the molecular ion intensity of pretilachlor in the TD split-type DBDI system with helium, N_2_, and air as the discharged gas. By comparing the intensity of pretilachlor’s molecular ions obtained in the case of the three different carrier gases, it could be seen that the target analyte was directly ionized without a carrier gas (open air) and the intensity reached approximately 78.7% of that obtained under helium (Figure 6d). As a result, it was verified that this method is available for on-site detection without the helium and N_2_ that are used as discharged gas in traditional DBDI [44].

### 3.5. On-Site Non-Destructive Analysis

**Figure 7:**
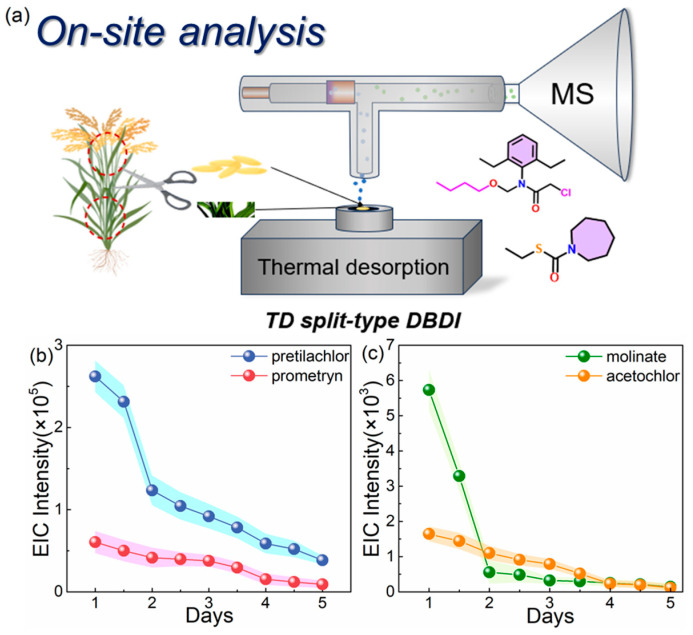
(**a**) Schematic diagram of the on-site non-destructive testing process. The changes in the EICs’ intensities of a piece of leaf (length of 1 cm) from a rice plant in the paddy field within a week after being sprayed with different herbicides: (**b**) 1 ppm pretilachlor and 1 ppm prometryn; (**c**) 1 ppm molinate and 1 ppm acetochlor (20 μL).

With the development of technology, portable miniaturized mass spectrometers are becoming more prevalent, which might be more suitable for further on-site or on-line measurements [45,46,47]. On-site detection as a novel method of rapid inspection has become increasingly popular, as it makes spot checks easy for the supervisor. In order to verify the performance of the TD split-type DBDI ionized system applied in on-site testing, we randomly selected parts of rice plants in a paddy field in Ningbo region and sprayed them with different concentrations of standard herbicides to monitor the residues of herbicides daily. For fast and convenient detection, a non-destructive analysis method was adopted, in which the spike or leaf of rice was picked directly from the rice and cut into pieces with length of 1 cm, which did not affect the normal growth of the rice plant (Figure 7a). The sample fragments were directly placed on the heating platform for detection without any pretreatment, and the relative ion signal’s intensity was recorded in each test. For better carrying out the experiments, we set the day of spraying herbicide as the first day. It is worth mentioning that the following experiments were carried out in the open air to demonstrate that the method is suitable for field detection.

As shown in Figure 7b,c, the rice was grown in a paddy field that was sprayed with a pretilachlor solution (1 ppm, 20 μL), a prometryn solution (1 ppm, 20 μL), a molinate solution (1 ppm, 20 μL), and an acetochlor solution (1 ppm, 20 μL), which were far below the spraying concentrations in general operation. Samples were placed on the heating platform with a desorption temperature of 160 °C, and the EIC’s intensity gradually decreased over time. Importantly, the testing samples from the rice 5 days after spaying exhibited strong enough intensities in the EIC for determining the existence of herbicides (intensity: 10^2^–10^5^), reflecting the efficient dissociation and ionization of this system and the persistence of herbicide residues (Figure 7b,c). The intensity of the EICs of pretilachlor and molinate decreased dramatically, which indicated the fast volatilization of pretilachlor and molinate on the surface of the rice samples within 2 days. On the contrary, prometryn and acetochlor gave regular volatilization ascribed to the relatively high vapor pressure (2 × 10^−6^ mmHg, 2.8 × 10^−5^ mmHg), which demonstrated that the saturated vapor pressure had significant influence on the detection of solid substances. Importantly, a remarkable S/N (≥10) for the herbicides above could be obtained even 10 days after spraying, revealing excellent sensitivity in this application situation. However, for properly understanding the working cooperativity of the system, the rice samples were prepared by the method above and detected by the system without heating, while the herbicide residues could not be effectively detected after 2 days (Figures S10 and S11). In all samples sprayed with acetochlor and pretilachlor, the necessary intensities (<50) of the EICs of the relative ions could not be detected on the third day, which might result from complete volatilization of the herbicides on the surface. In addition, it suggests an explanation that internal herbicides permeated into the rice sample did not volatilize due to their low vapor pressures (Table S2). Therefore, the programmable heat played a positive role in accelerating the volatilization and gasification of analytes.

In addition, the TD split-type DBDI ionized system showed good capability for the detection of herbicides and exhibited potential for on-site detection. The detection performance of this method used for on-site non-destructive analysis was investigated. As showed in Table S4, LODs of 1.5–18 μg/kg with RSDs of less than 20% and R^2^ values of 0.9941–0.9995 for these herbicides were achieved, demonstrating that this detection method met the requirements of the national standards and could be used to assess samples’ compliance. Given this performance, the method is suitable for field testing to establish a risk assessment method, which is expected to minimize the impact of herbicides on human health and the environment. However, due to the complexity of open environments, the quantitative ability for the direct analysis of solids needs continuous improvement.

## 4. Conclusions

In this work, we present a TD split-type DBDI with separation of the sample and the carrier gas inlets to achieve efficient ionization and transfer of the analyte for quantitative detection of a broad class of herbicides in rice. Under optimization of the working parameters, the efficiency of transfer and ionization of the analyte were improved to achieve the quantitative detection of herbicides in solutions and residues spiked in an actual matrix, which provided the advantages of high sensitivity, high speed, and high selectivity by gasification of the target analytes. The programmable heating platform ensured efficient gasification and transfer of the target analyte, avoiding the notable matrix effect. Furthermore, when it was applied for the simultaneous detection of different kinds of herbicides, results with good sensitivity, stability, and reliability were achieved. Moreover, the ionized system exhibited a relatively acceptable performance using air as the discharged gas (open air) compared with helium. On basis of this finding, the TD split-type DBDI system produced great performance in on-site analysis by monitoring herbicide residues in the growth stage, which made the inner residues volatilize as much as possible to obtain lower LODs. This method shows great potential for the supervision of the food safety market, achieving non-destructive detection of crops anytime and anywhere. For better technical transformation, we can further explore the analysis of other pesticides via the TD split-type DBDI system and improve the adaption of this system. This finding may provide new insights into the determination of pesticides’ emergence and quality assessments of rice.

## Figures and Tables

**Table 1:** Analytical results of the six herbicides spiked in rice samples by the new method.

Analyte	L.R. (μg/kg)	Regression Equation	R^2^	MRLs (μg/kg)	LOQ (μg/kg)	LOD (μg/kg)	RSD
Prometryn	1–100	y = 3113.67x + 309.72	0.9998	20	1	0.3	14.2%
Molinate	10–80	y = 246.62x − 422.59	0.9871	100	10	3	9.8%
Alachlor	10–100	y = 23.13x + 56.9	0.9968	50	10	3	14.8%
Acetochlor	10–100	y = 51.51x − 11.31	0.9990	50	10	3	8.9%
Pretilachlor	1–100	y = 4521.93x − 504.59	0.9999	10	1	0.3	11.9%
Butachlor	20–100	y = 32.43x − 90.90	0.9957	100	20	6	7.6%

## Data Availability

The original contributions presented in the study are included in the article/Supplementary Material, further inquiries can be directed to the corresponding author.

## Supplementary Materials

The following supporting information can be downloaded at: https://www.mdpi.com/article/10.3390/foods13081280/s1. Figure S1: Mass spectra of molecular ions and fragmentation ions of pretilachlor. Figure S2: Mass spectra of molecular ions and fragmentation ions of molinate. Figure S3: Mass spectra of molecular ions and fragmentation ions of alachlor. Figure S4: Mass spectra of molecular ions and fragmentation ions of acetochlor. Figure S5: Mass spectra of molecular ions and fragmentation ions of prometryn. Figure S6: Mass spectra of molecular ions and fragmentation ions of butachlor. Figure S7: Standard calibration curves for six standard herbicides in methanol at various concentrations of 0.1–100 ppb: (a) prometryn, (b) molinate, (c) alachlor, (d) acetochlor, (e) pretilachlor and (f) butachlor. Figure S8: The EIC peaks for parent ions with three consecutive injections and corresponding mass spectra of (a,b) alachlor (500 μg/kg), (c,d) acetochlor (500 μg/kg), (e,f) butachlor (1000 μg/kg) and (g,h) molinate (500 μg/kg). Figure S9: The EIC peaks of fragmentation ions for six herbicides with three consecutive injections. Figure S10: The EIC and mass spectra of a piece (length of 1 cm) of leaf from rice samples grown naturally for two days after spraying with pretilachlor solution (1 ppm, 20 μL) and prometryn solution (1 ppm, 20 μL), which detected by the TD split-type DBDI ionized system without heating. Figure S11: The EIC and mass spectra of a piece of leaf (length of 1 cm) from rice samples grown naturally for two days after spraying with molinate solution (1 ppm, 20 μL) and acetochlor solution (1 ppm, 20 μL), which detected by the TD split-type DBDI ionized system without heating. Table S1: Preparation method for matrix-contained standard samples with different concentrations. Table S2: Basic information (physicochemical properties and ions) about six herbicides. Table S3: The linear range (L.R.), regression equations, linear correlation coefficients (R^2^), and limit of detection (LOD) of standard herbicides in the methanol. Table S4: Analytical results of herbicides by on-site non-destructive analysis.
