# Supplementary material for: A Facile Determination of Herbicide Residues and Its Application in On-Site Analysis
Source: Foods. 2024 Apr 22;13(8):1280. doi: 10.3390/foods13081280 (PMC11049070; doi:10.3390/foods13081280)
Supplement: Supplementary file 1 [file foods-13-01280-s001.zip › foods-2966329-supplementary.pdf]

Supplementary materials

A facile determination of herbicide residues and its application in on-site analysis

Yifei Sun [1, 2, 3], Yan Tang [1, 3], Zetao Chen [1, 3], Miaoxiu Ge [1, 3], Wei Xiong [1, 3, *] and Luhong Wen [1, 3, *]

[1] The Research Institute of Advanced Technology, Ningbo University, Ningbo, 315211, P. R. China
[2] Faculty of Electrical Engineering and Computer Science, Ningbo University, Ningbo, 315211, P. R. China
[3] China Innovation Instrument Co., Ningbo, 315100, P. R. China
* Correspondence: xiongwei@nbu.edu.cn (W. X.); wenluhong@nbu.edu.cn (L. W.)

Table S1. Preparation method for matrix-contained standard samples with different concentrations.

Samples	Added standard solution concentration (ppm)	Added standard solution volume (μL)	Matrix additive concentration (μg/kg)
Crushed rice (10 g)	0.1	100	1
	1	20	2
	1	50	5
	1	100	10
	10	20	20
	10	30	30
	10	40	40
	10	50	50
	10	60	60
	10	80	80
	10	100	100

Table S2. Basic information (physicochemical properties and ions) about six herbicides.

Compound	Saturated vapor pressure	Ion form	Measured *m/z* value	Quantitative ions *m/z*
prometryn	2×10^{-6} mmHg	$[M+H]^+$	242.2	200.1
molinate	3.1×10^{-9} mmHg	$[M+H]^+$	188.1	126.1
alachlor	2.2×10^{-5} mm Hg	$[M+H]^+$	270.1	238.1
acetochlor	2.8×10^{-5} mmHg	$[M+H]^+$	270.1	224.1
pretilachlor	5.2×10^{-8} mmHg	$[M+H]^+$	312.2	252.2

butachlor	2.0×10^{-6} mmHg	$[M+H]^+$	312.2	238.2

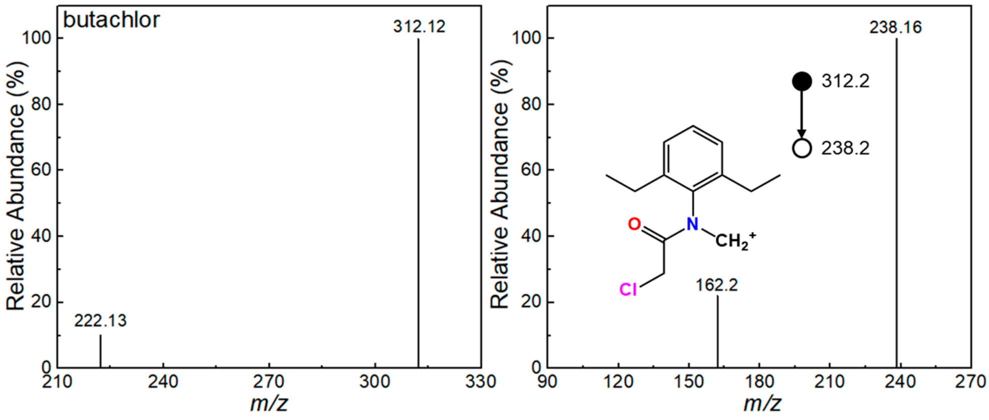

Figure S1. Mass spectra of molecular ions and fragmentation ions of pretilachlor.

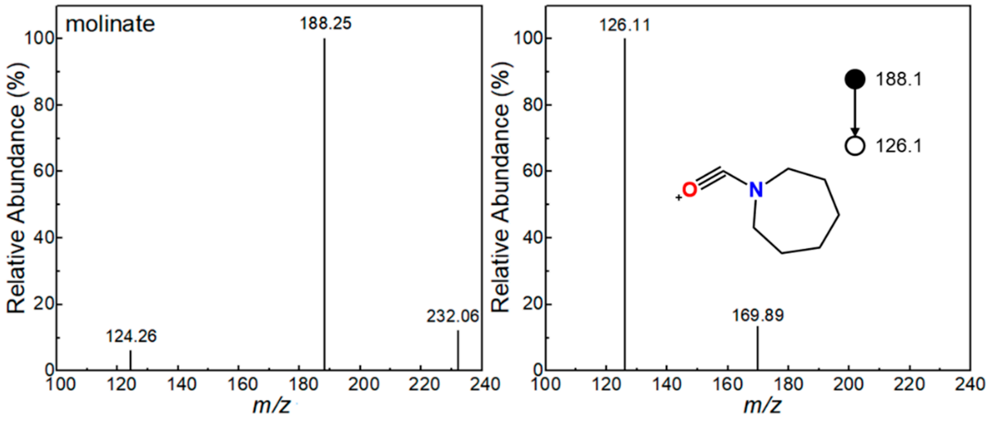

Figure S2. Mass spectra of molecular ions and fragmentation ions of molinate.

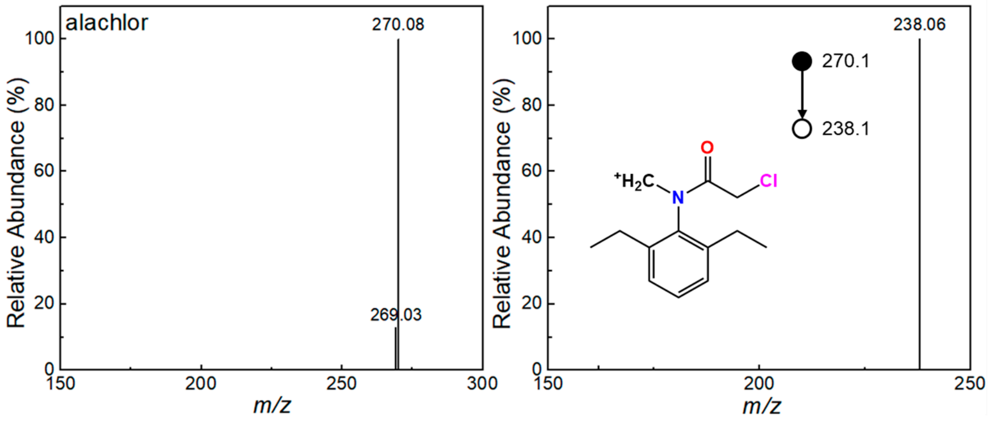

Figure S3. Mass spectra of molecular ions and fragmentation ions of alachlor.

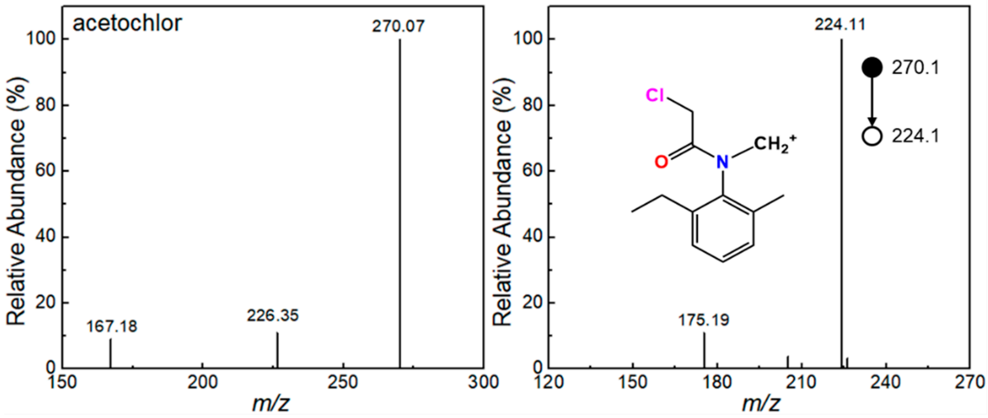

Figure S4. Mass spectra of molecular ions and fragmentation ions of acetochlor.

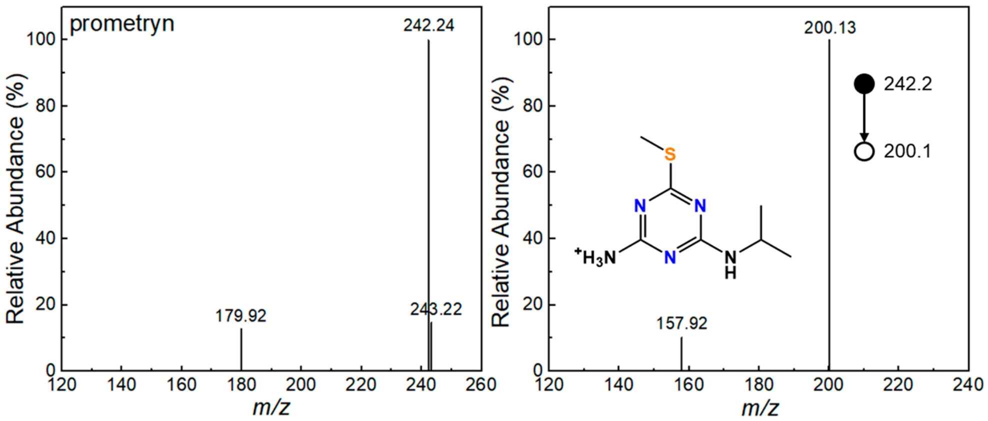

Figure S5. Mass spectra of molecular ions and fragmentation ions of prometryn.

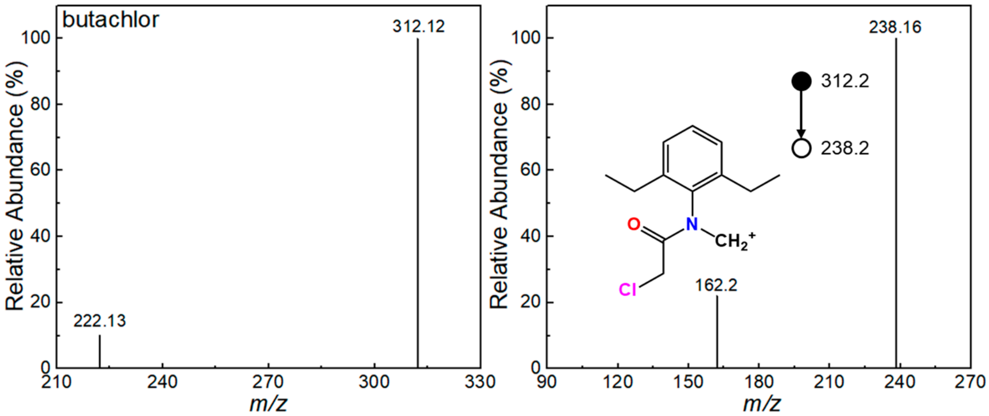

Figure S6. Mass spectra of molecular ions and fragmentation ions of butachlor.

Table S3. The linear range (L.R.), regression equations, linear correlation coefficients (R^2), and limit of detection (LOD) of standard herbicides in the methanol.

Analyte	L.R. (ppb)	Regression equation	R^2	LOQ (ppb)	LOD (ppb)
prometryn	0.1-100	y = 5324.71x – 286.24	0.9931	0.1	0.03
molinate	5-100	y = 275.74x + 127.64	0.9954	5	1.5
alachlor	5-100	y = 33.28x + 36.09	0.9915	5	1.5
acetochlor	5-100	y = 55.95x + 356.88	0.9856	5	1.5
pretilachlor	0.1-100	y = 5358.99x + 110.83	0.9980	0.1	0.03
butachlor	5-100	y = 38.13x + 61.84	0.9964	5	1.5

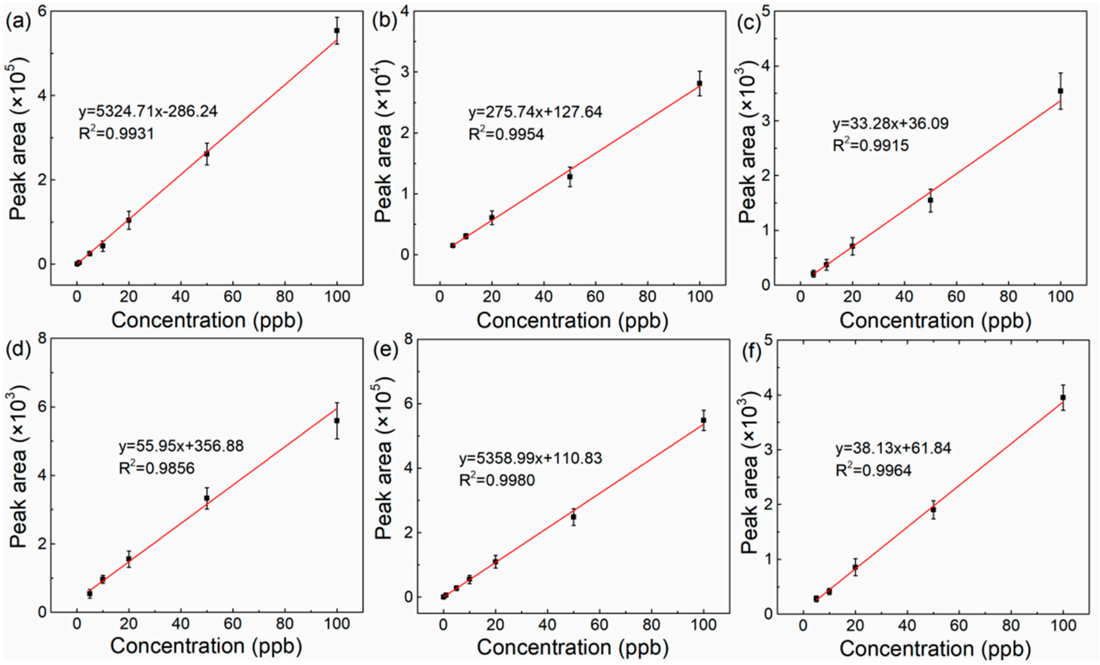

Figure S7. Standard calibration curves for six standard herbicides in methanol at various concentrations of 0.1-100 ppb: (a) prometryn, (b) molinate, (c) alachlor, (d) acetochlor, (e) pretilachlor and (f) butachlor.

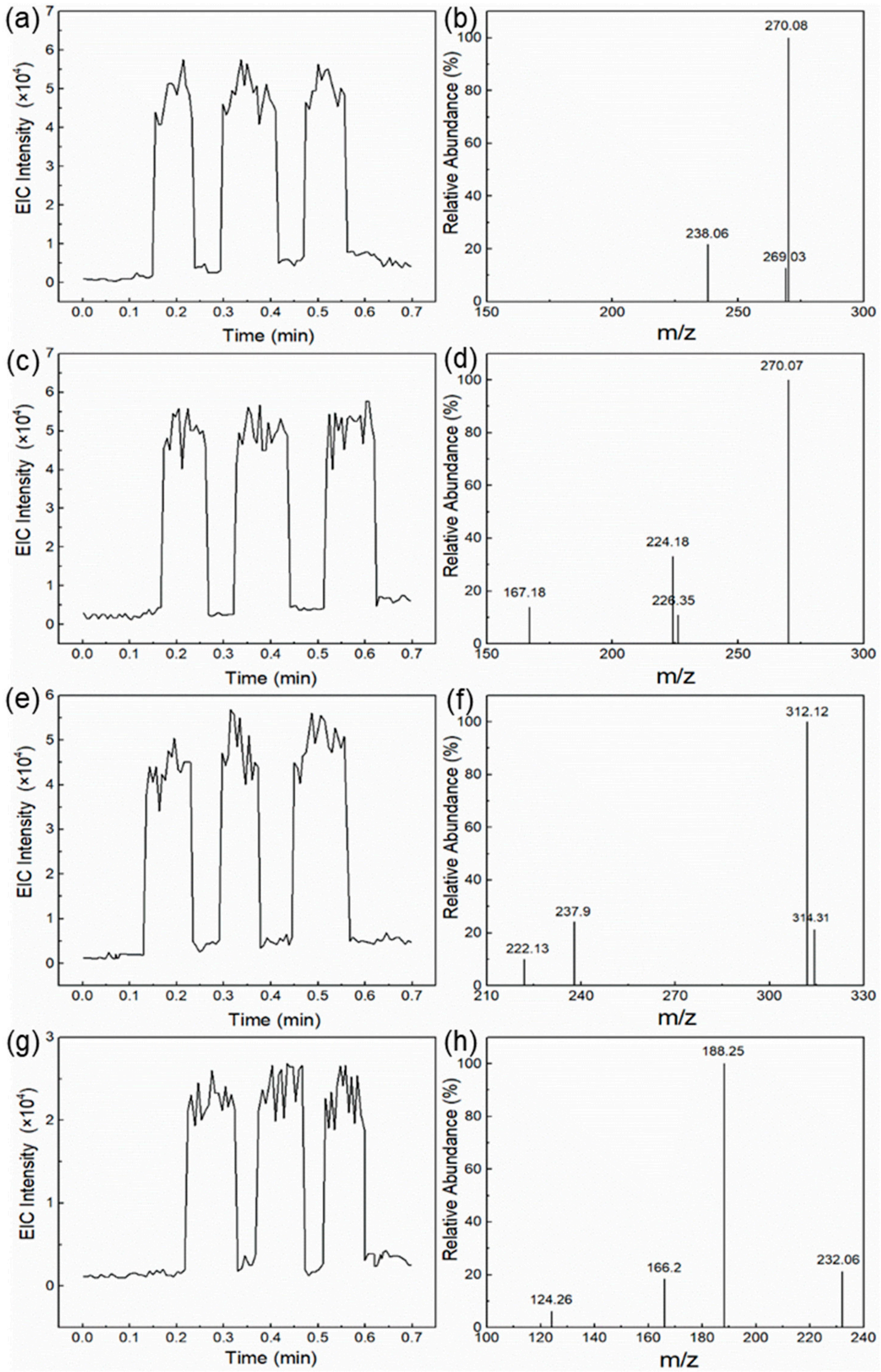

Figure S8. The EIC peaks for parent ions with three consecutive injections and corresponding mass spectra of (a, b) alachlor (500 μg/kg), (c, d) acetochlor (500 μg/kg), (e, f) butachlor (1000 μg/kg) and (g, h) molinate (500 μg/kg).

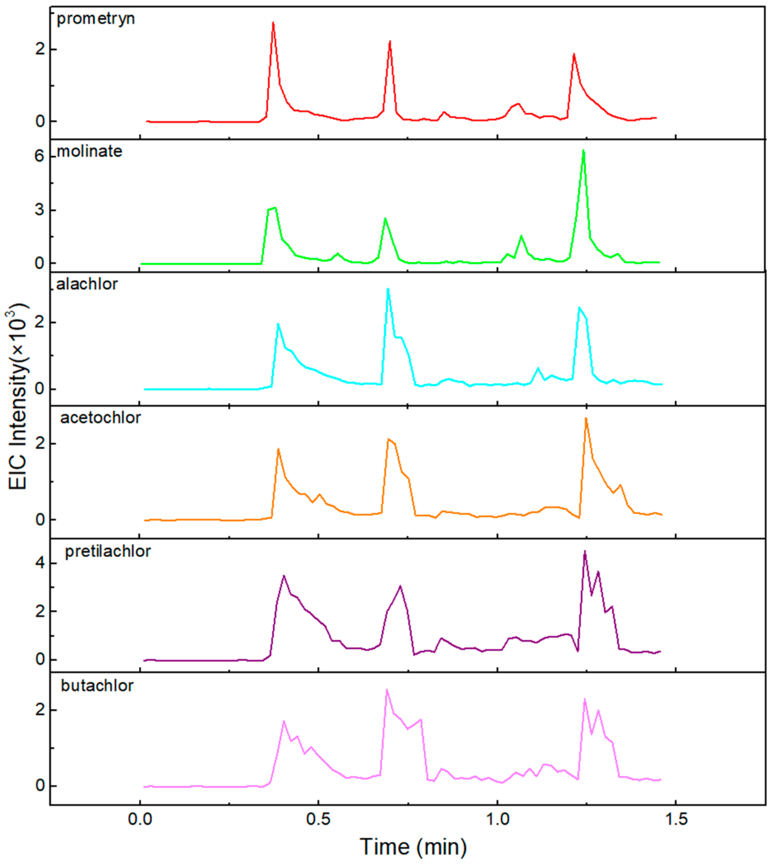

Figure S9. The EIC peaks of fragmentation ions for six herbicides with three consecutive injections.

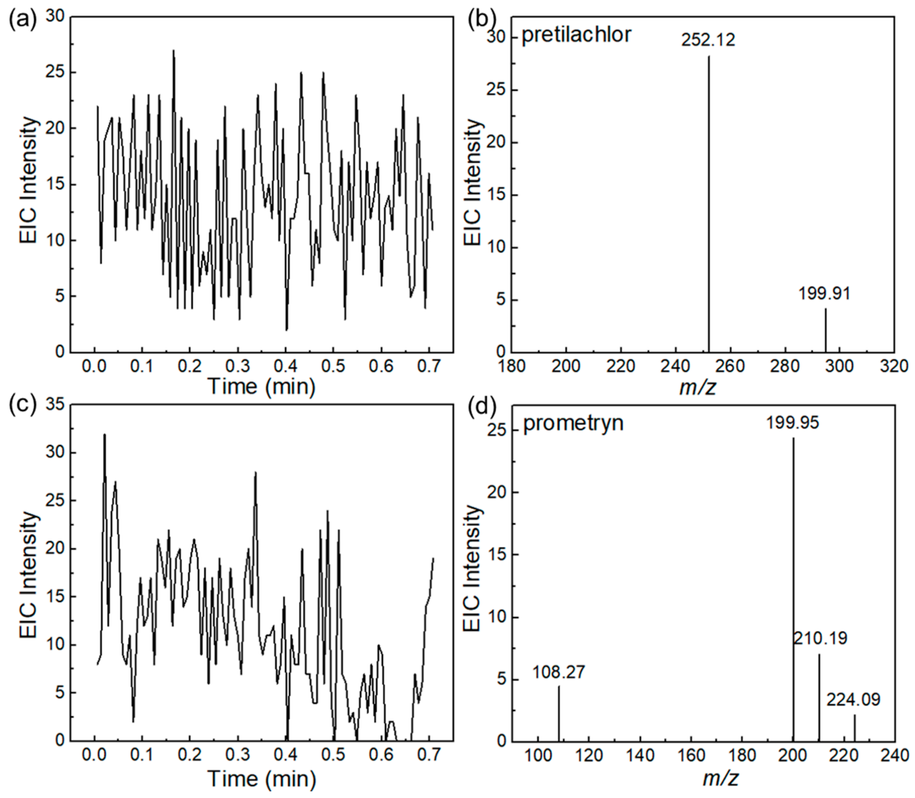

Figure S10. The EIC and mass spectra of a piece (length of 1 cm) of leaf from rice samples grown

naturally for two days after spraying with pretilachlor solution (1 ppm, 20 μL) and prometryn solution (1 ppm, 20 μL), which detected by the TD split-type DBDI ionized system without heating.

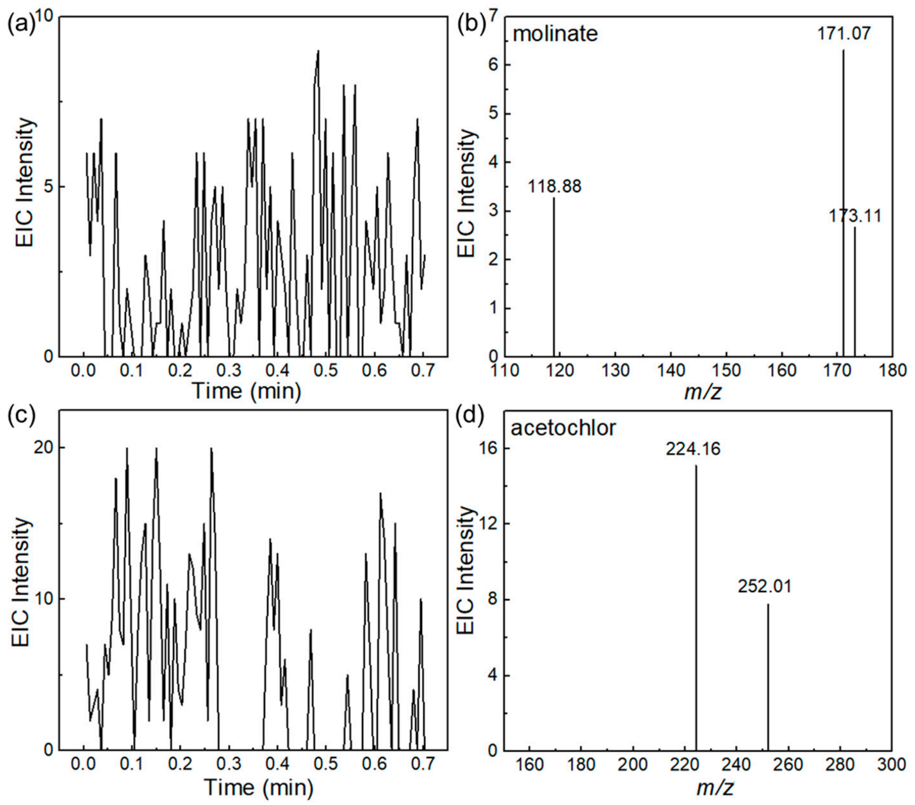

Figure S11. The EIC and mass spectra of a piece of leaf (length of 1 cm) from rice samples grown naturally for two days after spraying with molinate solution (1 ppm, 20 μL) and acetochlor solution (1 ppm, 20 μL), which detected by the TD split-type DBDI ionized system without heating.

Table S4. Analytical results of herbicides by on-site non-destructive analysis.

Analyst	L.R. (μg/kg)	Regression equation	R^2	LOD (μg/kg)	RSD
pretilachlor	5-100	y = 365.452x + 17.647	0.9975	1.5	15.2%
prometryn	10-100	y = 370.4x - 117.6	0.9952	3	17.2%
molinate	50-200	y = 70.7154x - 216.65	0.9941	15	12.4%
acetochlor	60-200	y = 20.968x - 143.71	0.9995	18	13.5%
